# The Lipid Mediator Resolvin D1 Reduces the Skin Inflammation and Oxidative Stress Induced by UV Irradiation in Hairless Mice

**DOI:** 10.3389/fphar.2018.01242

**Published:** 2018-10-31

**Authors:** Priscila Saito, Cristina P. B. Melo, Renata M. Martinez, Victor Fattori, Talita L. C. Cezar, Ingrid C. Pinto, Allan J. C. Bussmann, Josiane A. Vignoli, Sandra R. Georgetti, Marcela M. Baracat, Waldiceu A. Verri, Rubia Casagrande

**Affiliations:** ^1^Laboratory of Oxidative Stress and Inflammation, Department of Pharmaceutical Sciences, Londrina State University, Londrina, Brazil; ^2^Laboratory of Pain, Inflammation, Neuropathy, and Cancer, Department of Pathology, Londrina State University, Londrina, Brazil; ^3^Department of Biochemistry and Biotechnology, Londrina State University, Londrina, Brazil

**Keywords:** resolvin, inflammation, oxidative stress, UVB irradiation, lipid mediator

## Abstract

UV irradiation-induced oxidative stress and inflammation contribute to the development of skin diseases. Therefore, targeting oxidative stress and inflammation might contribute to reduce skin diseases. Resolvin D1 (RvD1) is a bioactive metabolite generated during inflammation to actively orchestrate the resolution of inflammation. However, the therapeutic potential of RvD1 in UVB skin inflammation remains undetermined, which was, therefore, the aim of the present study. The intraperitoneal treatment with RvD1 (3-100 ng/mouse) reduced UVB irradiation-induced skin edema, myeloperoxidase activity, matrix metalloproteinase 9 activity, and reduced glutathione depletion with consistent effects observed with the dose of 30 ng/mouse, which was selected to the following experiments. RvD1 inhibited UVB reduction of catalase activity, and hydroperoxide formation, superoxide anion production, and gp91phox mRNA expression. RvD1 also increased the Nrf2 and its downstream targets NQO1 and HO-1 mRNA expression. Regarding cytokines, RvD1 inhibited UVB-induced production of IL-1β, IL-6, IL-33, TNF-α, TGF-β, and IL-10. These immuno-biochemical alterations by RvD1 treatment had as consequence the reduction of UVB-induced epidermal thickness, sunburn and mast cell counts, and collagen degradation. Therefore, RvD1 inhibited UVB-induced skin oxidative stress and inflammation, rendering this resolving lipid mediator as a promising therapeutic agent.

## Introduction

The skin is the largest organ of the human body and the main protection barrier of the organism against chemical, physical, and biological aggressors (Afaq et al., 2005; Fonseca et al., 2011; Khavkin and Ellis, 2011). External aggressors include exposure to UV irradiation, the main cause of skin damage. Acute exposure to UV irradiation may lead to a number of effects such as edema, sunburn, erythema, inflammation, and prolonged or chronic exposure can lead, for instance, to immunosuppression, premature aging, and skin cancer (Afaq et al., 2005; Fonseca et al., 2011; Tewari et al., 2013a; Martinez et al., 2015b).

The skin damage induced by UV irradiation occurs due to overproduction of reactive oxygen species (ROS), such as the superoxide anion (Ivan et al., 2014), consequently leading to depletion of endogenous antioxidant, such as reduced glutathione (GSH) (Zaid et al., 2007; Halliwell, 2009). The imbalance between generation and removal of free radicals in the body leads to a pro-oxidant state that can lead to cell damage, including cell death (Júnior et al., 2001). The UVB irradiation spectrum is considered the most damaging and harmful UV irradiation for the human skin (Afaq et al., 2005; Hupel et al., 2011; Figueiredo et al., 2014), because its main consequence is direct DNA damage, mainly in keratinocytes. In addition, exposure to UVB irradiation activates the skin immune system components, triggering inflammatory response through the release of inflammatory mediators such as cytokines that will orchestrate the inflammatory response (Bowden, 2004; Hildesheim et al., 2004; Oliveros et al., 2009; Maverakis et al., 2010; Balogh et al., 2011). Considering the synergistic effect of the production of ROS and inflammatory mediators, the improvement of the endogenous antioxidant system and the resolution of inflammation become promising approaches to prevent and treat UVB irradiation-induced skin damage (Fonseca et al., 2010; Serhan, 2014).

Resolvin D1 (RvD1) is a bioactive metabolite generated in response to inflammation by enzymatic conversion of docosahexaenoic acid (DHA) (Seki et al., 2010). The RvD1 belongs to the group of lipid mediators that play important roles in the resolution phase of inflammation (Chan and Moore, 2010; Recchiuti and Serhan, 2012; Moro et al., 2016). These lipid mediators have been a major focus in recent years due to their anti-inflammatory and pro-resolution abilities in various disease models. Their actions include reduction of neutrophil chemotaxis, induction of neutrophil apoptosis, chemoattraction of non-phlogistic macrophages, reduction of dendritic cell migration to the lymph nodes and IL-12 production, and increasing macrophage-mediated clearance of cell debris, apoptotic cells, and invading microorganisms (Serhan et al., 2008; Serhan, 2014).

Studies have shown that RvD1 is able to inhibit the inflammatory response and promote the resolution of inflammation by reducing the production of the pro-inflammatory cytokines TNF-α and IL-1β in mouse models of acute lung injury induced by lipopolysaccharides (LPS). In addition, the ability of RvD1 to reduce oxidative stress in lung injury was demonstrated through increased transcription of the gene encoding the enzyme heme-oxygenase 1 (HO-1) (Wang et al., 2014). Other studies have demonstrated the reduction of inflammatory responses in asthma and acute lung injurie with the use of RvD1, in addition to suppression chemokine production and oxidative stress induced by cigarette smoke extract (Haworth and Levy, 2007; Rogerio et al., 2012; Hsiao et al., 2013).

These results make reasonable to envisage that RvD1 has, in principle, the potential to reduce inflammation and oxidative stress in other disease conditions. However, there is no evidence on the effect of RvD1 in UVB irradiation-induced skin inflammation and oxidative stress, which we reason to be worthy investigating in the present study.

## Materials and methods

### Chemicals

Chemicals were obtained from the following sources: resolvin D1 from Cayman Chemical (Ann Arbor, Michigan, USA); brilliant blue R, reduced glutathione (GSH), hexadecyltrimethylammonium bromide (HTAB), *o*-dianisidine dihydrochloride, 5,5′-dithiobis (2-nitrobenzoic acid) (DTNB), nitroblue tetrazolium (NBT), and bisacrylamide from Sigma-Aldrich (St. Louis, MO, USA); tert-butyl hydroperoxide from Acros (Pittsburgh, PA, USA); tris from Amresco (Solon, OH, USA); ELISA kits for determination of cytokine from eBioscience (San Diego, CA, USA); and acrylamide, sodium dodecyl sulfate (SDS), platinum SYBRGreen, and superscript III kits from Invitrogen. All other reagents used were from pharmaceutical grade.

### Animals

Sex matched hairless mice (HRS/J) weighing 20–30 g were obtained from the University Hospital of Londrina State University under the following conditions: 12 h dark/12 h light cycle and 23 ± 2°C temperature. The mice were maintained with free access to water and food throughout the experiment. The animal protocol used in this study was approved by Animal Ethics Committee (CEUA process number 1447.2015.10) of the Londrina State University.

### Experimental protocol

Five mice per group were randomly assigned to six groups. The groups were: non-irradiated control treated with vehicle (saline), irradiated control treated with vehicle (saline), irradiated treated with RvD1 3 ng/mice, irradiated treated with RvD1 10 ng/mice, irradiated treated with RvD1 30 ng/mice, irradiated treated with RvD1 100 ng/mice.

Treatment doses of RvD1 were 3, 10, 30, and 100 ng/ mouse, via intraperitoneal administration, 1 h before and 7 h after the beginning of UV irradiation (Martinez et al., 2015a). Animals in the control groups received treatment with vehicle (saline) used in the dilution of the drug. The doses of RvD1 used in the treatments were selected based on the therapeutic effects of studies published in other disease models (Spite et al., 2009a; Hsiao et al., 2013; Wang et al., 2014) and on dose-response curves tested in the present study.

Based on the results obtained in the assays evaluating skin edema, GSH, MPO and MMP-9, one dose of RvD1 was selected to the following experiments quantitating oxidative stress and oxidative stress-related mRNA expression (catalase, hydropexide formation, and superoxide anion production, and qPCR to determine gp19phox, Nrf2, Nqo-1, and HO-1 mRNA expression), cytokine production (IL-1β, IL-6, IL-33, TNF-α, TGF-β, and IL-10), and skin tissue alterations (epidermal thickness, sunburn cell counts, mast cell counts, collagen degradation). The time points of sample collection after UVB irradiation and assays to be performed at each time point were determined in standardization experiments. Cytokine production, oxidative stress and antioxidant markers were evaluated at earlier time points than the tissue alterations since tissue alterations are consequences of mediator production (Campanini et al., 2013; Martinez et al., 2015b, 2017).

### Irradiation

The light source used in the experiments to induce oxidative stress and acute inflammatory process in hairless mice was a fluorescent UVB lamp model PHILIPS TL/12 40W RS (MEDICAL-NETHERLANDS). The lamp emits irradiation in the range of 270 to 400 nm with a peak emission at 313 nm. The dose of irradiation used to induce inflammation and oxidative stress was 4.14 J/cm^2^ (Campanini et al., 2013).

Mice were kept at a distance of 20 cm from the lamp as previously described (Campanini et al., 2013) and were irradiated simultaneously. The hairless mice were terminally anesthetized with 5% isoflurane (Abbott [Abbott Park, IL, USA]) 12 h after the end of UVB irradiation and the full thickness of the dorsal skins were removed for edema, MPO activity, MMP-9 activity and GSH assays and histology. Moreover, the hairless mice were anesthetized with 5% isoflurane, following by decapitation at 2 h for catalase and NBT assays, and 4 h for evaluation of production hydroperoxides, cytokines measurement and PCR after the end of UVB exposure and the dorsal skins were removed. Each parameter was evaluated at a specific time, which was previously determined (Campanini et al., 2013; Martinez et al., 2015b). The dorsal skin samples were collected and stored at −80°C until analysis. The samples collected by verification of cutaneous edema were weighed immediately after collecting and by histology were fixed in buffered formaldehyde.

### Skin edema

UV irradiation increases the permeability of the vascular endothelium causing edema (Dusting and Macdonald, 1995). In order to evaluate the skin edema associated with the inflammatory process, the dorsal skin samples were collected from the animals with the aid of a mold with a fixed area (5 mm diameter). Edema was expressed by the variation of skin weight between the different control and treated groups (Ivan et al., 2014).

### Myeloperoxidase (MPO) activity

Myeloperoxidase (MPO) activity was quantitated to be used as a marker of the leukocyte infiltrate (monocytes/macrophages and neutrophils) on the skin after UVB irradiation (Katiyar and Meeran, 2007).

The skin samples were collected in 50 mM phosphate buffer (pH 6.0) containing 0.5% hexadecyltrimethyl ammonium bromide (HTAB), homogenized with Tissue-Tearor (Biospec 985370) and centrifuged (16,100 g for 2 min at 4°C). Briefly, 30 μL of the resulting supernatant from each sample were mixed with 50 mM phosphate buffer (pH 6.0) containing 0.167 mg/mL o-dianisidine and 0.015% hydrogen peroxide. MPO activity was determined spectrophotometrically at 450 nm (EnSpire, Perkin Elmer). The MPO activity of the samples was obtained by comparison with the MPO activity of a standard neutrophil curve. The results were expressed in neutrophil numbers per mg of skin (Casagrande et al., 2006).

### Analyses of skin proteinase substrate-embedded enzymography

For the analyze of MMP-9, the polyacrylamide gel zymography technique with sodium dodecyl sulfate was applied (SDS), a method used to detect proteases. The analysis detects enzymes that degrade the gelatin present in the gel (Kim et al., 2007; Fonseca et al., 2011).

Skin samples were diluted (1: 4) and homogenized in 50 mM Tris-HCl buffer (pH 7.4) containing 10 mM calcium chloride (CaCl_2_) and 1% proteinase inhibitors (phenanthroline, phenylmethylsulfonyl fluoride and N—ethylmaleimide) with the aid of Tissue-Tearor (Biospec 985370). Thereafter, the homogenates were centrifuged (12,000 g, 10 min, 4°C) twice. Supernatant aliquots (25 μL) were mixed with 5 μL of 0.1 M Tris-HCl (pH 7.4) containing 20% glycerol, 4% SDS, and 0.005% xylene cyanol and applied on electrophoresis gel (13.5% acrylamide and 0.025% gelatin). After electrophoresis, the gels were washed for 1 h with 2.5% Triton X-100 under constant shaking, incubated overnight in 0.05 M Tris-HCl (pH 7.4) and 0.01M CaCl_2_ at 37°C. The next day, the gels were stained with brilliant blue R and destaining in 20% acetic acid. The zones of enzymatic activity were detected as regions of negative staining against a dark background. The proteolytic activity was analyzed quantitatively by comparing the results of the samples of the treated animals with the controls not treated by the Image J program (NIH, Bethesda, MD, USA) (Onoue et al., 2003; Casagrande et al., 2006).

### Quantification of endogenous antioxidant reduced glutathione (GSH)

Skin samples were diluted in 0.02M EDTA and triturated using Tissue-Tearor (Biospec 985370). Whole homogenates were treated with 50% trichloroacetic acid. The mixture was then centrifuged at 2,700 g for 10 min at 4°C. The supernatant was removed and recentrifuged at 2,700 g for a further 15 min at 4°C. The final supernatant was removed for analysis. For the assay, the reaction mixture contained 50 μL of the sample supernatant, 100 μL of 0.4 M Tris buffer pH 8.9 and 5 μL of a 1.9 mg/mL solution of 5,5'-dithio-bis- (2-nitrobenzoic acid; DTNB) in methanol. The absorbance was determined in a spectrophotometer (EnSpire, Perkin Elmer) after 5 min of incubation at 405 nm. The standard curve was prepared with 0 to 150 μM GSH. The results were expressed in as μM of GSH per mg of skin (Srinivasan et al., 2007).

### Levels of the of endogenous antioxidant catalase (CAT)

The method is based on the concentration decay of hydrogen peroxide (H_2_O_2_) which is directly proportional to the absorbance decrease at 240 nm. The difference in absorbance per unit time is the measure of catalase activity (Aebi, 1984).

Skin samples were homogenized in 500 μL of 0.02M EDTA using the Tissue-Tearor homogenizer (Biospec 985370). The homogenate was centrifuged at 2,700 g for 10 min at 4°C twice. The determination of CAT activity on skin was performed on microplate by addition of 10 μL sample, 160 μL 1M Tris-HCl buffer with 5 mM EDTA pH 8.0, 20 μL deionized water, and 20 μL 200 mM H_2_O_2_. A white was included for each sample prepared with 10 μL of the sample supernatant, 180 μL of 1M Tris-HCl buffer with 5 mM EDTA pH 8.0, and 20 μL of deionized water. The rate at which H_2_O_2_ is reduced by the action of CAT was evaluated by decreasing the absorbance value by the difference between the initial reading and reading 30 s after the addition of 200 mM H_2_O_2_. The reading was performed on a microplate spectrophotometer (Enspire, Perkin Elmer) at 240 nm with a temperature maintained at 25°C. The catalase values were expressed as unit of CAT/ mg skin/ minute (Aebi, 1984).

### Assay for lipid peroxidation (LPO)

Lipid peroxidation is one of the most important organic expressions of oxidative stress (Yagi, 1987). Oxidation of lipids was measured by the formation of hydroperoxides, which are the primary products in lipid peroxidation (Linggnert et al., 1979).

The hydroperoxide production was evaluated by the chemiluminescence method previously described (Martinez et al., 2015b). The method is based on the determination of the chemiluminescence initiated by the tert-butyl hydroperoxide (Gonzalez Flecha et al., 1991).

The dorsal skin samples were homogenized in 800 μL of phosphate buffer (pH 7.4) using a Tissue-Tearor (BIOSPEC 985370) and centrifuged at 700 g for 2 min at 4°C. For the assay, 250 μL of the supernatant was diluted to 1,730 μL of reaction medium (120 mM KCl, 30 mM phosphate buffer pH 7.4) and 20 μL of 3 mM tert-butyl hydroperoxide. The reading was conducted in a β–counter Beckman® LS 6000SC (FULLERTON, CA, USA) in a non-coincident counting for 30 s with a response range between 300 and 620 nm. The experiment was performed in the darkin order to avoid vial phosphorescence activated by light at 30°C for 2 h. The results were measured in counts per min (cpm) per mg of skin.

### Evaluation of the production of superoxide anion (${\text{O}}_{2}^{\cdot -}$)

Superoxide anion assay was performed through the nitroblue tetrazolium assay (NBT). Skin samples were homogenized with Tissue-Tearor (BIOSPEC 985370) in 0.02 M EDTA and centrifuged (2,000 g for 20 s at 4°C). For the reaction, the supernatant (50 μL) was incubated in microplates for 1 h. Then the supernatant was removed and NBT (1 mg/mL) added to the fixed cells. After 15 min, the NBT was carefully removed and 20 μL of 100% methanol was added to the precipitate to settle. The compound formed by the reduction of NBT (formazan) was solubilized with 120 μL of 2M KOH and 140 μL of dimethylsufoxide (DMSO). The reduction of NBT to formazan was measured in a microplate spectrophotometer reader (ENSPIRE, PERKIN ELMER) at 620 nm and the results are presented as optical density (OD) per 10 mg of skin (Campanini et al., 2013).

### Cytokine measurement

The dorsal skin samples were homogenized in saline (500 μL) with Tissue-Tearor (Biospec 985370) and centrifuged at 2,000 g for 15 min at 4°C. The supernatants were used to quantify the cytokines IL-1, IL-6, IL-33, TNF-α, TGF-β, and IL-10 aby enzyme-linked immunosorbent assay (ELISA) according to manufacturer's instructions (eBioscience). The results were obtained by comparing the optical densities at 490 nm of the samples with the densities of the respective cytokine standard curves (Verri et al., 2008).

### Quantitative polymerase chain reaction (QPCR)

Followed the method described elsewhere (Campanini et al., 2013; Martinez et al., 2016b). Briefly, samples were homogenized in trizol reagent for total RNA extraction. The purity of total RNA was measured spectrophotometrically, and the wavelength absorption ratio (260/280 nm) was between 1.8 and 2.0 for all preparations. Reverse transcription of total RNA to cDNA was carried out using a Superscript III kit (Invitrogen) and oligo (dT)12–18 primers. Real-time PCR (qPCR) was performed with Platinum SYBRGreen kits (Invitrogen) in a 50 μL reaction volume following the manufacturer's cycling conditions. Melting curve analysis was performed in order to verify that only one product was amplified. Samples with more than one peak were excluded. qPCR was performed in a LightCycler Nano Instrument (Roche). The relative gene expression was measured using the comparative 2^−−(ΔΔ*Cq*)^ method. The expression of gyceraldehyde-3-phosphate dehydrogenase (Gapdh) mRNA was used as a control for tissue integrity in all samples. The primers used were gp91^phox^, sense: 5′-AGCTATGAGGTGGTGATGTTAGTGG-3′, antisense: 5′- CACAATATTTGTACCAGACAGACTTGAG-3′; Nrf2, sense: 5′-TCACACGAGATGAGCTTAGGGCAA-3′, antisense: 5′-TACAGTTCTGGGCGGCGACTTTAT-3′; Nqo-1, sense: 5′-TGGCCGAACACAAGAAGCTG-3′, antisense: 5′-GCTACGAGCACTCTCTCAAACC-3′; HO-1, sense: 5′-CCCAAAACTGGCCTGTAAAA-3′; antisense: 5′-CGTGGTCAGTCAACATGGAT-3′; and Gapdh sense: 5′-ATGACATCAAGAAGGTGGTG-3, antisense: 5′-CATACCA- GGAAATGAGCTTG-3′;

### Skin histologic evaluation

The dorsal skin samples were collected in formol 10%, fixed in paraformaldehyde 4%, dehydrated in ascending concentrations of ethanol, cleared in xylene, embedded in paraffin and sectioned to a thickness of 5 μm. The sections were stained with hematoxylin and eosin, toluidine blue and Masson's trichrome stain.

The sections stained with H & E were examined using light microscopy at 40x magnification for determination of epidermal thickness (Deng et al., 2015) and a 100x magnification for counting the number of sunburn cells (Schwarz et al., 1995). For mast cell count, the sections were stained with toluidine blue and analyzed under light microscopy at 40x magnification. Both analyses were done with the software Infinity Analyze (Lumenera® Software). The sections stained with masson's trichrome were examined using light microscopy at a magnification of 10x to visualize changes in collagen fibers by analyzing the intensity of the blue coloration in the dermal areas of the skin exposed to UVB with the aid of the Image J software (NIH) (Song et al., 2016).

### Statistical analysis

The bars in the results indicate the mean values ± standard error of the mean (SEM) of 5 mice per group per experiment and are representative of two separate experiments. Data were statistically analyzed by ANOVA followed by Tukey's *t*-test. Statistical analyses were performed using GraphPad Prism 7 software (GraphPad Software Inc., San Diego, CA, USA). Results were considered significantly different when *p* < 0.05.

## Results

### Resolvin D1 (RvD1) reduces UVB irradiation-induced skin edema and MPO activity

**Figure 1:**
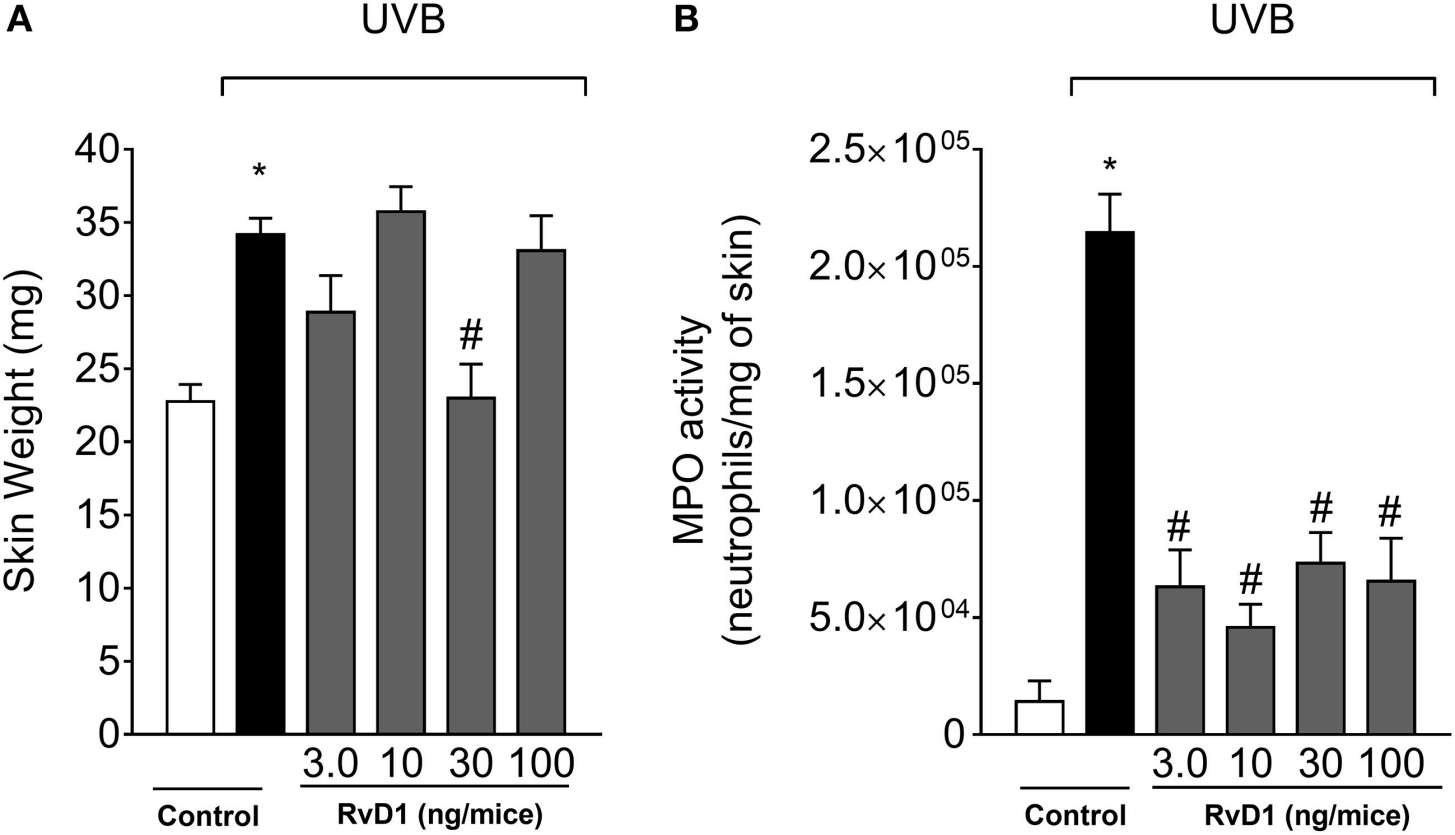
RvD1 reduces UVB irradiation-induced skin edema and MPO activity. The skin inflammation was determined in samples collected 12 h after the end of irradiation. **(A)** Skin edema and **(B)** MPO activity. Bars represent means ± SEM of 5 mice per group and are representative of two separate experiments. Statistical analysis was performed by one-way ANOVA followed by Tukey's test. [*p < 0.05 compared to the non-irradiated control group; ^#^*p* < 0.05 compared to the irradiated control group (vehicle)].

The anti-inflammatory action of RvD1 was first assessed by the edema assay and MPO activity (neutrophil marker). Skin edema was inhibited by RvD1 treatment only by the dose of 30 ng/mice (Figure 1A). On the other hand, all four doses of RvD1 showed a similar inhibition of MPO activity (Figure 1B). Based on the result obtained in edema, the dose of 30 ng/mouse was chosen for the next assays. Receptor expression in the target cellular population affects the efficacy of pro-resolution lipids as observed for aspirin-triggered lipoxin A4 (ATLA4). ATLA4 has dose-dependent effect over leukocyte chemoattraction. Nevertheless, the genetic induction of lipoxin receptor ALXR/FPR2 in myeloid cells enhances the activity of ATLA4 in a manner that 10 ng achieves the same efficacy of 1μg of ATLA4 (Chiang et al., 2005) Despite the wide range of doses tested, the effect of RvD1 over UVB irradiation was not dose-dependent. It is likely that further investigation on delivery route, formulation and time of treatment may improve the efficacy of RvD1 by reaching the right cellular target at the best time point and dose.

### RvD1 reduces UVB irradiation-induced increase of epidermal thickness and apoptosis of keratinocytes

**Figure 2:**
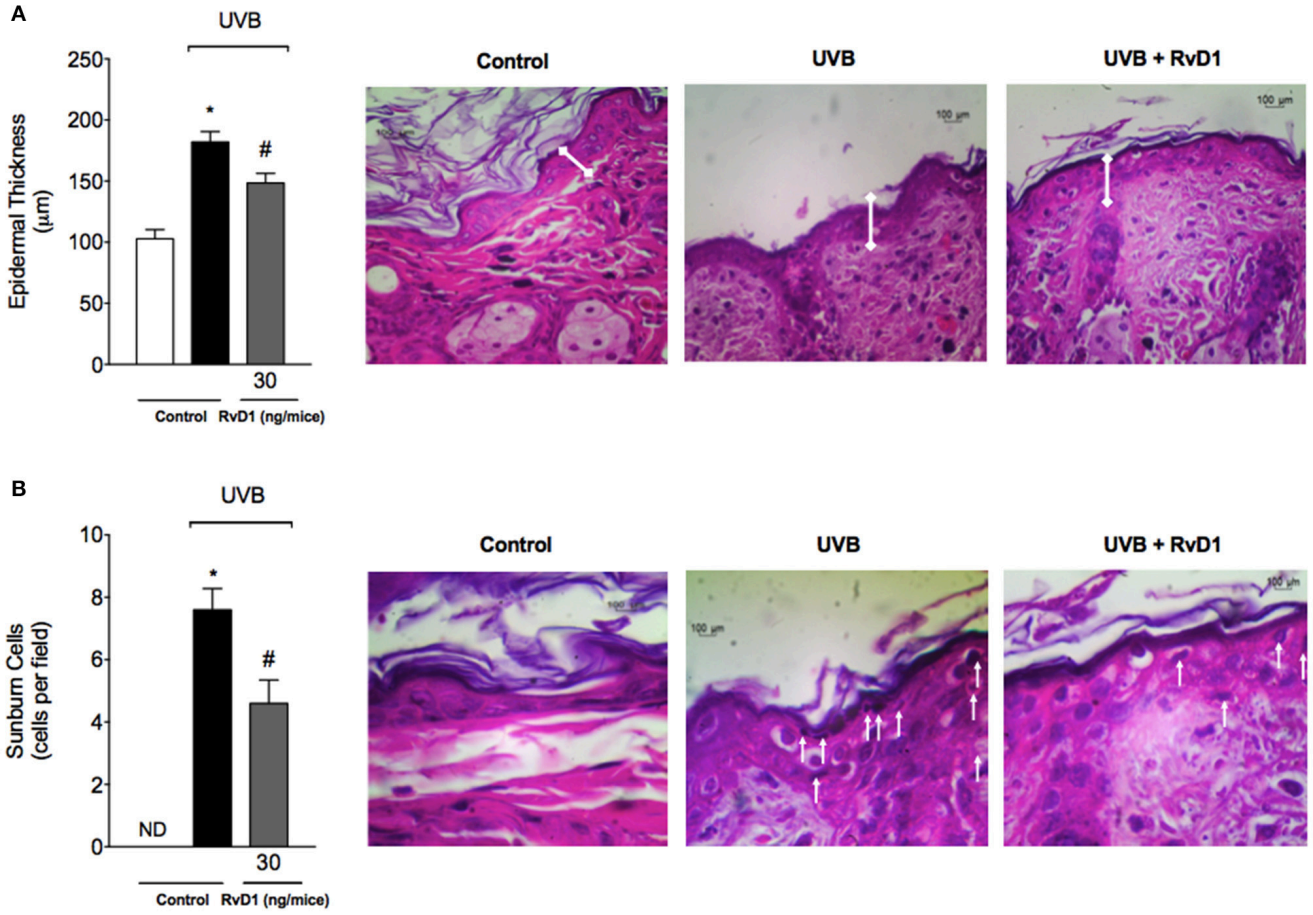
RvD1 reduces UVB irradiation-induced increase of epidermal thickness and sunburn cell counts. Epidermal thickness and sunburn cell counts were evaluated using hematoxylin and eosin staining (H & E) in skin samples collected 12 h after the end of irradiation. Epidermal thickness (μm) **(A)** and the number of sunburn cells **(B)**. No sunburn cells were detected in the negative control group, which was indicated as ND. The sections stained with H & E were examined using light microscopy at 40x **(A)** magnification and 100x **(B)**. Bars are representative of two separate experiments and represent means ± SEM of 5 mice per group per experiment. Statistical analysis was performed by one-way ANOVA followed by Tukey's test. [*p < 0.05 compared to the non-radiated control group; ^#^*p* < 0.05 compared to the radiated control group (vehicle)].

Epidermal thickness is used as a quantitative parameter to assess inflammation (Martinez et al., 2015b). Measurement of hematoxylin and eosin stained tissue sections indicated that dorsal skin epidermal thickness was significantly increased following UVB irradiation in the irradiated control group compared to the non-irradiated control group. In contrast, epidermal hypertrophy was significantly reduced by 57.48% compared with irradiated control group when mice were treated with RvD1 (Figure 2A).

One of the consequences of acute exposure to UV radiation is the activation of apoptosis of epidermal keratinocytes, which are defined as shrunken cells within the epidermis that exhibit eosinophilic cytoplasm and condensed nucleus, and called sunburn cells (Bayerl et al., 1995). UV radiation induced an increase on sunburn cell counts, which was inhibited by 39.47% compared with irradiated control group by RvD1 treatment (Figure 2B).

### RvD1 reduces UVB irradiation-induced increase of mast cells

**Figure 3:**
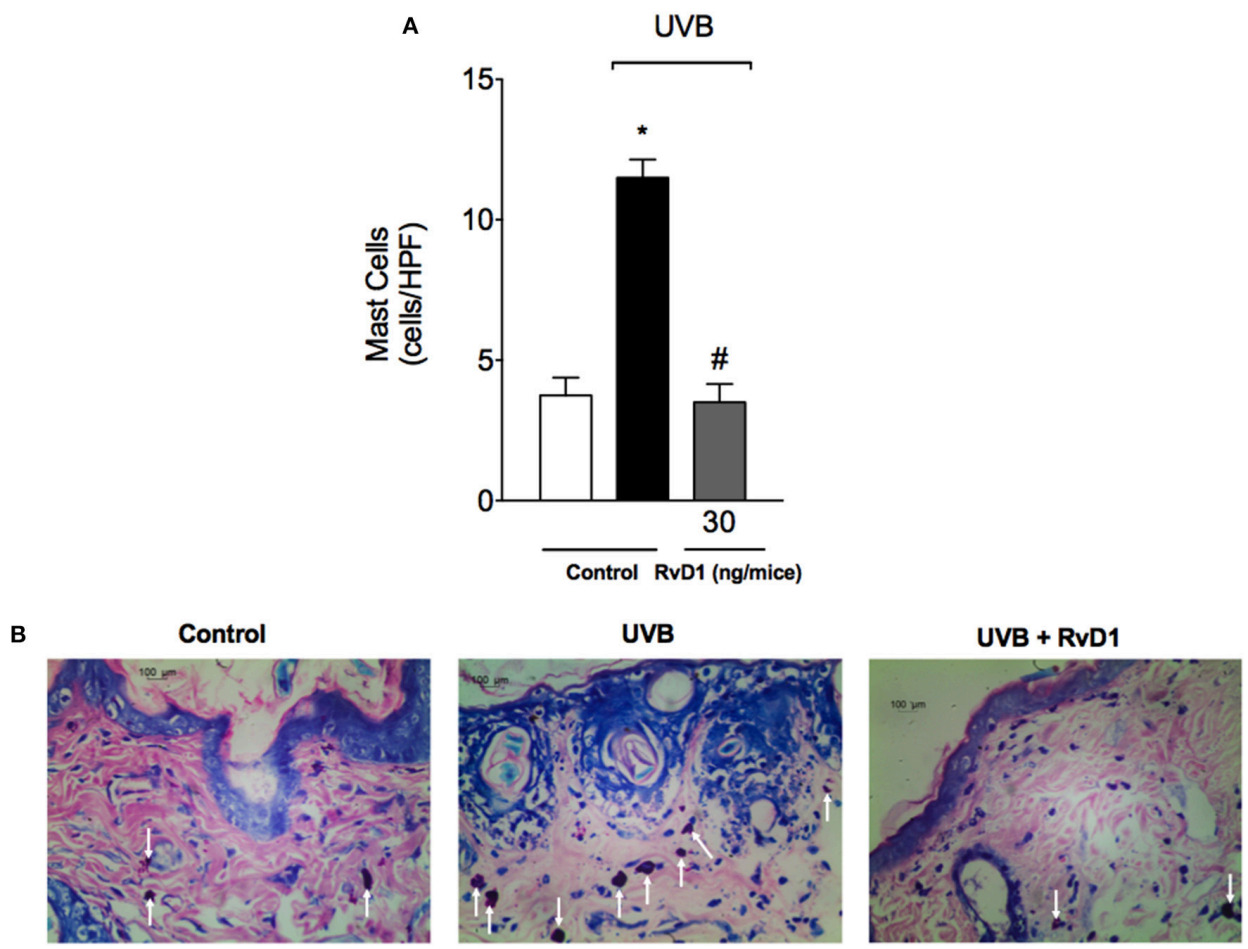
RvD1 reduces UV irradiation-induced increase of mast cell counts. Mast cell counts were evaluated using blue toluidine in skin samples collected 12 h after the end of irradiation. The number of mast cells **(A)** in the sections stained with blue toluidine were examined using light microscopy at 40x magnification **(B)**. Bars are representative of two separate experiments and represent means ± SEM of 5 mice per group per experiment. Statistical analysis was performed by one-way ANOVA followed by Tukey's test. [*p < 0.05 compared to the non-radiated control group; ^#^*p* < 0.05 compared to the radiated control group (vehicle)].

UVB irradiation induces a significant increase in the number of mast cells in the skin as well as their degranulation releasing varied pro-inflammatory mediators (Hart et al., 2001). In this study, treatment with RvD1 inhibited the UVB irradiation increase of mast cells counts (Figure 3).

### RvD1 reduces UVB irradiation-induced skin MMP-9 activity and damage of collagen fiber

**Figure 4:**
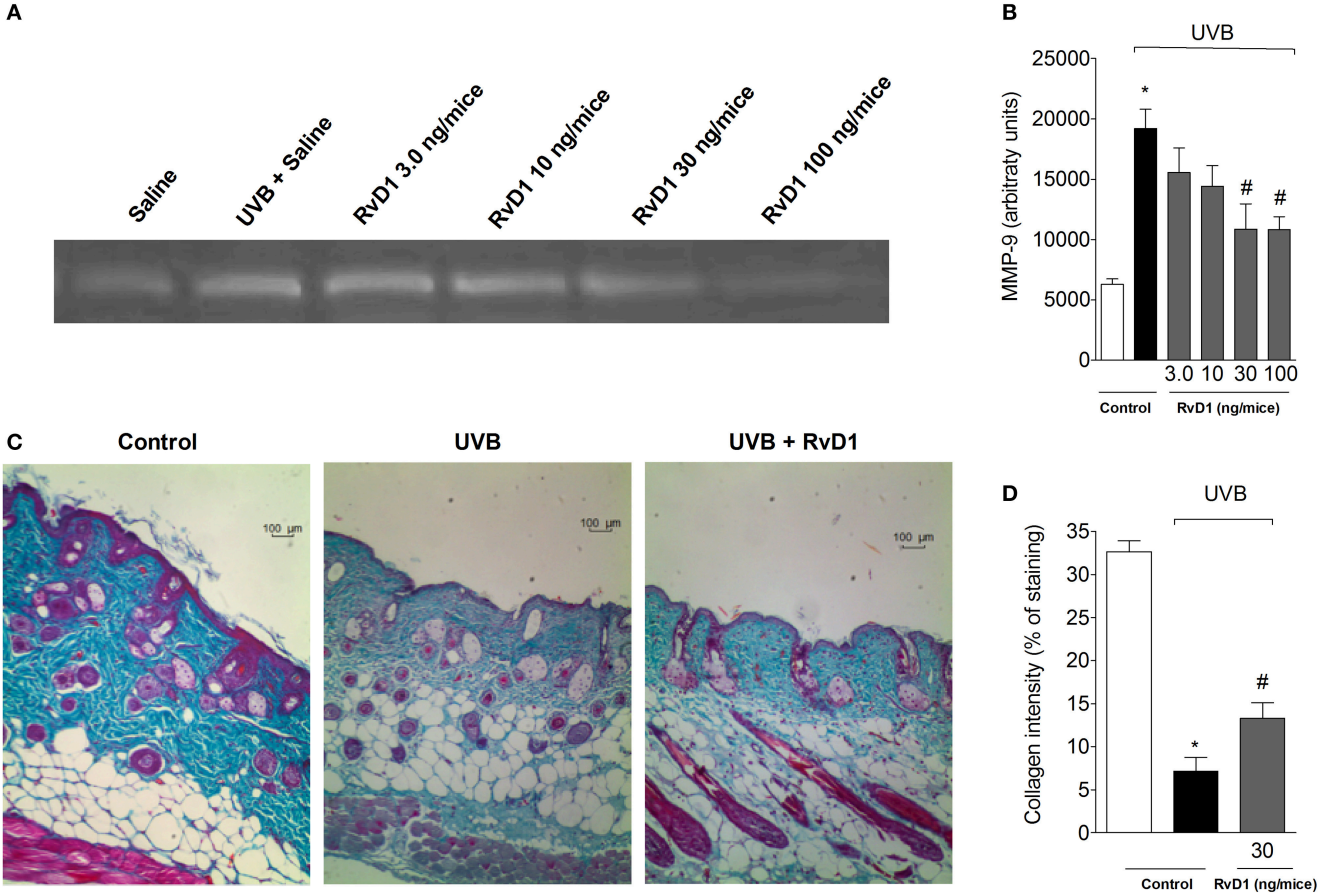
RvD1 inhibits UVB irradiation-induced increase of MMP-9 activity and damage to skin collagen fiber of hairless mice. The MMP-9 activity and collagen fiber formation were determined in samples collected 12 h after the end of irradiation. **(A)** Representative image of gelatin zymography and **(B)** MMP-9 activity. Collagen fiber formation was evaluated using Masson's trichrome staining. Collagen fiber intensity and bundles shown in blue were analyzed by Image J Program (10x magnification) **(C)**. Collagen intensity **(D)**. Bars are representative of two separate experiments and represent means ± SEM of 5 mice per group per experiment. Statistical analysis was performed by one-way ANOVA followed by Tukey's test. [*p < 0.05 compared to the non-radiated control group; ^#^*p* < 0.05 compared to the radiated control group (vehicle)].

MMP-9 is a gelatinase involved in the degradation of the elastic fiber network and collagen matrix, thus, it is involved in tissue remodeling and collagen fiber density (Jenkins, 2002). MMP-9-induced damage to the collagenous matrix of the skin is one of the hallmarks of photoaging and non-melanoma skin cancer (Brennan et al., 2003; Grady et al., 2007). Treatment with RvD1 reduced the UVB irradiation-induced activity of MMP-9 in a dose-dependent manner with the peak of RvD1 activity obtained with the dose of 30 ng/mouse. The doses of 30 and 100 ng/mouse of RvD1 reduced the activity of MMP-9 (Figures 4A,B).

The tissue sections were subjected to Masson's trichrome staining in order to determine changes in collagen fiber density in the dermal areas of the UVB-exposed dorsal skin (Song et al., 2016). Notably, the collagen fibers stained in blue in the group pretreated with RvD1 30 ng/mice showed lower levels of damage in collagen fiber compared with irradiated group (Figures 4C,D).

### RvD1 inhibits UVB irradiation-induced cytokine production

**Figure 5:**
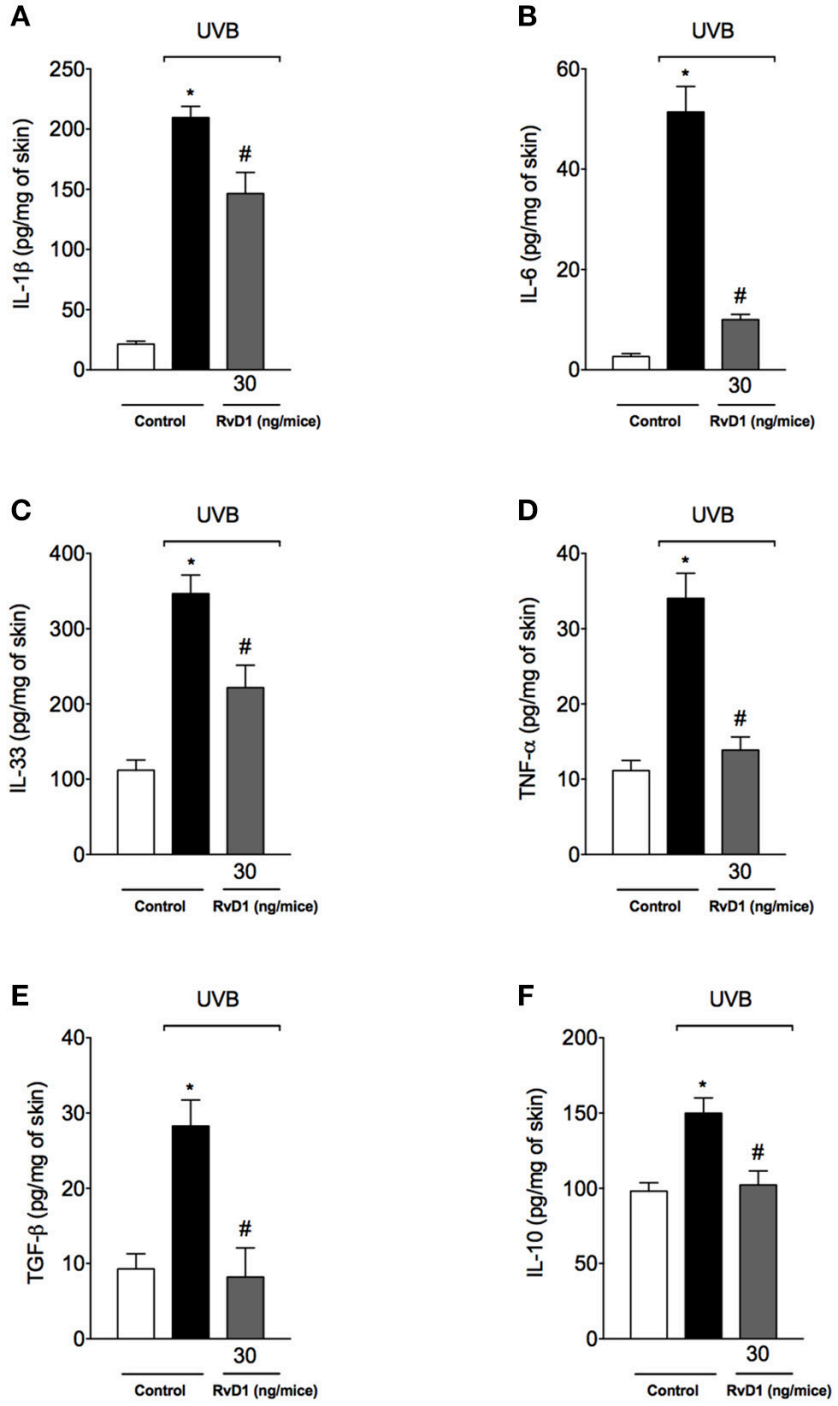
RvD1 inhibits UVB irradiation-induced cytokine production **(A)** IL-1β **(B)** IL-6 **(C)** IL-33 and **(D)** TNF-α and inhibits anti-inflammatory cytokines production **(E)** TGF-β and **(F)** IL-10. Cytokines were determined in skin samples collected 4 h after the end of irradiation. Bars represent means ± SEM of 5 mice per group and are representative of two separate experiments. Statistical analysis was performed by one-way ANOVA followed by Tukey's test. [*p < 0.05 compared to the non-radiated control group; ^#^*p* < 0.05 compared to the radiated control group (vehicle)].

Cytokines such as IL-1β, IL-6, IL-33, and TNF-α are involved in many cellular and tissue alterations in UVB irradiation, which include the recruitment of neutrophils that produce ROS and MMP-9, thus, with implications on inflammation and tissue remodeling (Garcia et al., 1999; Witko-Sarsat et al., 2000; Robinson et al., 2004; Verri et al., 2006; Walz and Cayabyab, 2017). Cytokines also affect vascular permeability, inducing tissue edema (Joosten et al., 2006; Zarpelon et al., 2013; Staurengo-Ferrari et al., 2018). Other cytokines such as TGF-β and IL-10 limit inflammation and orchestrate tissue repair (Verri et al., 2006; Penn et al., 2012). Therefore, cytokines were quantitated. UVB irradiation induced the production of pro-inflammatory (IL-1β, IL-6, IL-33, and TNF-α) and anti-inflammatory (TGF-β and IL-10) cytokines in hairless mice skin, which were inhibited by RvD1 treatment (Figures 5A–F). Therefore, RvD1 treatment reduced the cytokine production irrespectively whether these were pro-inflammatory or anti-inflammatory cytokines.

### RvD1 reduces UVB irradiation-induced oxidative stress, and enhances mRNA expression of genes involved in antioxidant response and skin antioxidants

**Figure 6:**
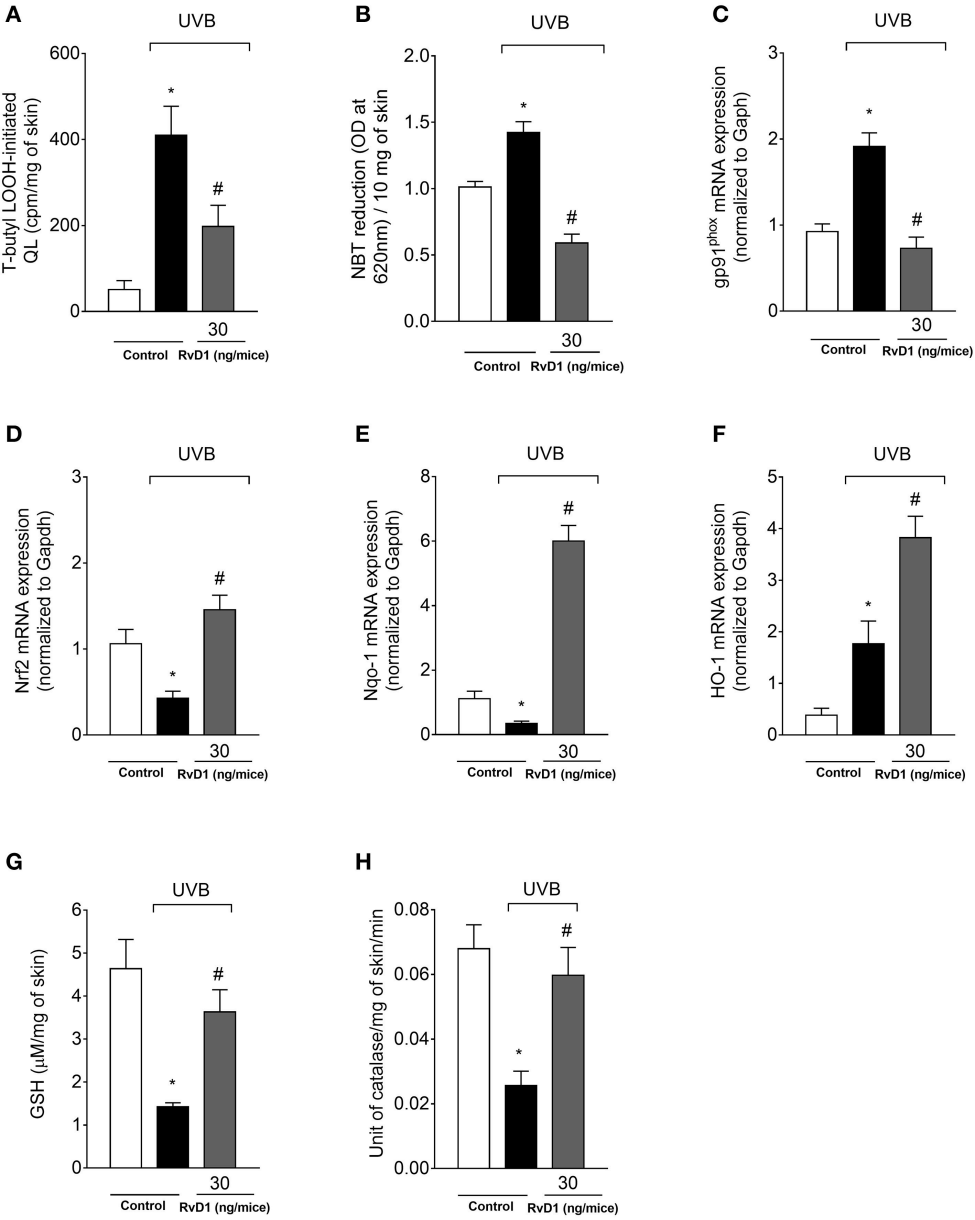
RvD1 inhibits UVB irradiation-induced oxidative stress, mRNA expression of oxidative stress-related genes and antioxidant depletion. Lipid peroxidation **(A)** was determined by t-butyl lipid hydroperoxides (LOOH)-initiated chemiluminescence (QL) at 4 h, superoxide anion production **(B)** was determined by nitroblue tetrazolium (NBT) reduction assay at 2h; gp91^phox^, Nrf2 **(D)**, Nqo-1 **(E)**, and HO-1 **(F)** mRNA expression **(C)** were determined by quantitative polymerase chain reaction (qPCR) at 4 h after the end of irradiation; GSH levels **(G)** and CAT activity **(H)** were determined at 12 h and 2h after the end of irradiation, respectively. Bars represent means ± SEM of 5 mice per group per experiment and are representative of two separate experiments. Statistical analysis was performed by one-way ANOVA followed by Tukey's test. [*p < 0.05 compared to the non-radiated control group; ^#^*p* < 0.05 compared to the radiated control group (vehicle)].

UVB irradiation increased the hydroperoxide and superoxide anion production in the skin of irradiated control group compared to non-irradiated control group. Treatment with RvD1 at the dose of 30 ng/mouse reversed this effect by reducing LOOH and superoxide anion production (Figures 6A,B). The NADPH oxidase is an important source of superoxide anion during inflammation (Anrather et al., 2006), and UVB irradiation caused an increase of the NADPH oxidase subunit gp91^phox^ mRNA expression, which was also reduced by RvD1 (Figure 6C) corroborating the data on superoxide anion production (Figure 6B).

GSH, NQO-1, and HO-1 are downstream targets of Nrf2 (Loboda et al., 2016). We observed that UVB irradiation decreased Nrf2 and Nqo-1 mRNA expression and an increase on HO-1 mRNA expression in the skin at 4 h. RvD1 treatment inhibited the reduction of Nrf2 mRNA expression, not only reversed the down-modulation of Nqo-1, but increased its mRNA expression, and also further enhanced HO-1 mRNA expression in the skin (Figures 6D,E,F).

The effects of RvD1 were determined by reduced glutathione (GSH) quantitation and catalase activity. The dose of UVB irradiation used in the experiment was able to significantly reduce the endogenous antioxidant GSH and catalase in the irradiated control group compared to the non-irradiated control group. The treatment with 30 ng/mice of RvD1 inhibited the reduction of GSH levels following UVB irradiation (Figure 6G). In the GSH assays, a bell-shaped curve was observed, corroborating the adequality of the chosen dose. Catalase activity was reduced by the UVB irradiation and RvD1 inhibited this reduction (Figure 6H).

## Discussion

UV irradiation causes a number of adverse biological effects in the skin, such as premature aging and skin cancer (Afaq et al., 2005; Fonseca et al., 2011; Tewari et al., 2013b; Martinez et al., 2015a). The deleterious effect of UV irradiation in the skin, in particular UVB (280–315 nm), occurs due to excessive free radical production and direct DNA damage (Fernández-García, 2014). ROS are directly involved in the induction of the inflammatory process and oxidative stress, because they stimulate the secretion of cytokines (Ivan et al., 2014) and the depletion of endogenous antioxidants (Zaid et al., 2007; Halliwell, 2009). Recently, we demonstrated for the first time that lipoxin A4, a pro-resolution lipid mediator derived from the arachidonic acid, reduces UVB irradiation-induced skin inflammation and oxidative stress (Martinez et al., 2018). However, there was no evidence on the therapeutic effect of a pro-resolving lipid mediators derived from DHA metabolism on UVB skin inflammation and oxidative stress. In the present study, we demonstrated that RvD1 reduced the skin inflammation and oxidative stress caused by UVB irradiation by reducing the production of cytokines and ROS resulting in diminished skin cellular infiltrate, keratinocyte apoptosis, and collagen degradation. Thus, RvD1 presents a pronounced inhibition of UVB irradiation detrimental effects suggesting its therapeutic potential.

RvD1 inhibited UV-induced skin edema and thickening of the epidermis. These data involve a complex range of cellular events. The acute inflammatory process triggered by exposure to UV irradiation causes specific cellular events, including increased permeability of the vascular endothelium, impairment of lymphatics function, infiltration of polymorphonuclear leukocytes, activation of inflammatory macrophages, lymphocytes, and mast cells that release pro-inflammatory molecules at the site of the lesion, which consequently lead to skin edema, and proliferation of keratinocytes and epidermal cells (Cotran and Collins, 2009). In line with the mechanisms of UVB skin edema and RvD1 effects, RvD1 attenuates pulmonary edema in a model of lipopolysaccharide (LPS)-induced acute lung injury by reducing occludin and zona occludin-1 tight junction proteins deterioration (Xie et al., 2013) and vascular injury-induced neointimal hyperplasia (Miyahara et al., 2013). Therefore, RvD1 may reduce edema by targeting vascular permeability, but also cellular proliferation and recruitment.

UV irradiation induces the increase of mast cells in the dermis (Grimbaldeston et al., 2003) and keratinocyte damages causing modifications toward sunburn cell phenotype (Bayerl et al., 1995). The presence of mast cells in the dermis correlates directly with the degree of susceptibility to systemic immunosuppression induced by long term UVB, and suppression of the immune system allows UV-induced tumors not to be destroyed (Hart et al., 1998). At early time points, mast cells contribute to UV-induced skin inflammation. (Harvima and Nilsson, 2011). Mast cells are known to release a great variety of inflammatory mediators upon degranulation including leukotrienes (LT), histamine and prostaglandin E_2_ (PGE_2_) (Krystel-Whittemore et al., 2016). RvD1 treatment decreased the UVB-induced increase of mast cell counts in the present study. However, it is possible that RvD1 also inhibits the function of the mediators released by mast cells. For instance, RvD1 can inhibit LTD_4_- and histamine-induced conjunctival goblet cell secretion (Dartt et al., 2011)and intracellular calcium increase(Li et al., 2013), respectively. Further, RvD1 inhibits 48/80-induced mast cell degranulation and PGE_2_ production (Grabauskas et al., 2018), which could possibly involve the inhibition of the release of other mast cell mediators. Evidence using the mast cell deficient W/W^v^ mouse shows that mast cells and their derived prostaglandins contribute to UVB-induced skin edema, however, mast cells and their prostaglandins do not contribute to sunburn cell formation (Ikai et al., 1985). Sunburn cells are also used as markers of skin damage caused by UVB. When UV irradiation exceeds the protective response threshold of keratinocytes, these cells undergo apoptosis and die. The reduction in the number of sunburn cells indicates an increase in the photoprotection of keratinocytes. Histopathological analysis, showed that RvD1 treatment decreased the number of sunburn cells compared to the irradiated control group.

Neutrophils have the potential to increase the damage caused by UV irradiation, as they are able to release a variety of substances that are harmful to cells and tissue such as ROS (Garcia et al., 1999; Robinson et al., 2004) and serine proteases that contribute to the alternative processing of TNF-α and IL-1β (Meyer-Hoffert and Wiedow, 2011). It has been reported that RvD1 reduced MPO activity in liver injury model induced by carbon tetrachloride (CCl4) (Chen et al., 2016). In addition, in the acute cigarette smoke-induced lung inflammation model, RvD1 inhibits neutrophilic inflammation and increase neutrophil efferocytosis (Hsiao et al., 2013). We found that treatment with RvD1 decreased the recruitment of neutrophils induced by UVB irradiation, determined by MPO activity. Neutrophils also secrete MMP-9 (Meyer-Hoffert and Wiedow, 2011), a proteolytic enzyme that degrades extracellular matrix collagen during pathological processes such as photoaging (Kossodo et al., 2004). RvD1 treatment inhibited the increase of MMP-9 activity upon exposure to UVB irradiation, which lined up well with the reduction of collagen fibers degradation and production of TNF-α and IL-1β.

Treatment with RvD1 inhibited the increase of pro-inflammatory cytokines IL-1β, IL-6, IL-33, and TNF-α, and anti-inflammatory TGF-β and IL-10 induced by UVB exposure. UVB induces the activation of inflammasomes NLRC4, NLRP3, and AIM2 to activate caspase-1 to cleave pro-IL-1β to its active and secreted form, IL-1β (Feldmeyer et al., 2007; Sollberger et al., 2015; Hung et al., 2017). However, despite the contribution of caspase-1 to keratinocyte apoptosis, this event is independent on inflammasomes (Hasegawa et al., 2016). In addition to NLRP3 activation, UVB-induced damaged DNA also triggers the production of IL-6 (Feldmeyer et al., 2007; Sollberger et al., 2015). Mast cells degranulate upon UVB stimulus releasing TNF-α (Walsh, 1995) and targeting the TNFR1 receptor (Zhuang et al., 1999) and TNF-α mRNA half-life with pentoxifylline (Schwarz et al., 1997) showed that TNF-α plays a role in the development of sunburn cells (Schwarz et al., 1995). This evidence suggests that the RvD1 inhibition of TNF-α production may contribute to the reduced sunburn cell counts we observed. IL-1β, IL-6, TNF-α, and IL-33 have a role in recruiting leukocytes (Verri et al., 2010). For instance, UV induces the IL-33 expression by keratinocytes and dermal fibroblasts, which recruit dermal mast cells and skin-infiltrating neutrophils (Byrne et al., 2011). IL-33, IL-1β, and TNF-α also contribute to the development of inflammatory edema (Joosten et al., 2006; Zarpelon et al., 2013; Staurengo-Ferrari et al., 2018). Therefore, these data on inhibitory effect of RvD1 over UVB irradiation-induced pro-inflammatory cytokine production contribute to explain, at least in part, the reduction of skin inflammation (edema, mast cell counts, neutrophil recruitment, MMP-9 activity and collagen degradation).

RvD1 also reduced the production of IL-10 and TGF-β induced by UVB irradiation. IL-10 is an anti-inflammatory cytokine co-released with pro-inflammatory cytokines to achieve a fine tuning of inflammation (Verri et al., 2006). TGF-β as well, has an anti-inflammatory contribution and a tissue repair role since stimulates fibroblasts to produce collagen (Penn et al., 2012). These results also mean that UVB *per se* triggered the release of IL-10 and TGF-β as endogenous mechanisms to limit skin inflammation and orchestrate tissue repair. Thus, inhibiting IL-10 and TGF-β production may be a drawback effect of RvD1 and also an explanation for the partial effect of RvD1 on reestablishing collagen fiber density. However, there is also evidence that RvD1 delivery using neutrophil-derived nanoparticles accelerated keratinocyte healing and reduced inflammation, suggesting that selecting the adequate delivery system and even a mimetic delivery system may improve RvD1 therapeutic effects (Norling et al., 2011).

Cytokines such as TNF-α and IL-1β activate the phagocyte NADPH oxidase inducing the production of superoxide anion. This mechanism contributes to amplifying UVB tissue damage. However, it is also likely that the initial superoxide anion leaks from the mitochondria electron-transport chain Complex I and Complex III (Berneburg et al., 1999; Heck et al., 2003). Then, superoxide anion induces the production of cytokines including TNF-α and IL-1β triggering inflammation and amplifying tissue oxidative stress (Yamacita-Borin et al., 2015; Fattori et al., 2016). Cytokines chemoattract and activate phagocytes that will further produce superoxide anion (Garcia et al., 1999; Witko-Sarsat et al., 2000; Robinson et al., 2004) and RvD1 reduced gp91^phox^ mRNA expression and superoxide anion production, indicating a reduction of phagocyte NADPH oxidase (NOX2) activity. Accumulated ROS act on the cell biological membrane and the availability of polyunsaturated fatty acids regulates the lipid peroxidation process. High peroxidation levels are associated with harmful effects on biological systems, such as loss of fluidity, inactivation of membrane enzymes and receptors, and ion permeability increase, leading to cell membrane rupture. In addition, peroxidation products can damage DNA. LOOH, which are the primary products of lipid oxidation (Linggnert et al., 1979), were used as markers of oxidative stress in the present study, and it was found that RvD1 treatment reduced production of LOOH induced by UVB irradiation exposure.

RvD1 not only reduced oxidative stress, but also improved the antioxidant capacity of the skin after exposure to UVB irradiation by maintaining GSH and catalase activities at basal levels. GSH plays an important role in protecting skin cells against oxidative damage through the direct elimination of ROS or acting as a coenzyme of glutathione peroxidase (Zaid et al., 2007; Halliwell, 2009). Catalase is an antioxidant enzyme that converts hydrogen peroxide in water and molecular oxygen (Schallreuter et al., 1999; D'Orazio et al., 2013). The loss of UVB-mediated cell viability is associated with a notable decrease in endogenous antioxidant defenses, therefore weakening the cellular antioxidant defense system (Zaid et al., 2007; Halliwell, 2009). GSH expression is controlled Nrf2 (Harvey et al., 2009). The data from several studies also showed that RvD1 reduced oxidative stress. RvD1 increased the levels of GSH and HO-1 mRNA expression in carbon tetrachloride (CCl4)-induced acute liver injury model (Chen et al., 2016). Also, RvD1 induced GSH release in human chondrocytes obtained from osteoarthritis patients. Our finding showed that RvD1 increases redox status as indicated by enhanced GSH levels. These data are consistent with those of the literature indicating that RvD1 inhibits GSH depletion (Cox et al., 2015). Lipid peroxidation is an endpoint oxidative stress reaction that generates aldehydes, such as 4-hydroxy-trans-2-nonenal (HNE). In turn, HNE reacts with glutathione generating GS-HNE conjugates that induce leukocyte recruitment, superoxide anion formation and production pro-inflammatory lipid mediators. RvD1 was shown to reduce GS-HNE-induced inflammation indicating RvD1 also targets the inflammatory response triggered by newly generated inducers of inflammation (Spite et al., 2009b).

Nrf2 modulates the expression of antioxidant and detoxifying enzymes, known as phase II enzymes. In this group of enzymes, HO-1, Nqo-1, and catalase (Kobayashi and Yamamoto, 2005; Choi et al., 2013) are also included. Exposure to UVB irradiation increased HO-1 mRNA expression and decrease Nrf2 and Nqo-1 mRNA expression. HO-1 is an antioxidant and anti-inflammatory enzyme that is responsive to different stress conditions, including the inflammation process; in this sense HO-1 is essential to main cellular resistance during oxidative stress conditions. Enhanced HO-1 mRNA expression was associated with the resolution of inflammation and natural killer (NK) cell-mediated cytotoxicity, which may explain why HO-1 production increases while other antioxidant enzymes are inhibited (Listopad et al., 2007; Martinez et al., 2016a,b). Corroborating with the current understanding of Nrf2, HO-1, and NQO-1 activities, treatment with RvD1 increased Nrf2 mRNA expression, which resulted in an enhancement of Ho-1 and Nqo-1mRNA expression, and maintenance the GSH levels and catalase activity. In other models, RvD1 contributed to the protection from the deterioration of tight junction proteins in a model of acute lung injury induced by lipopolysaccharide in mice by inducing HO-1 expression (Xie et al., 2013); increased GSH levels and HO-1 mRNA expression in carbon tetrachloride (CCl4)-induced acute liver injury model (Chen et al., 2016); and inhalation of CO accelerates inflammation resolution by inducing RvD1-dependent activation of HO-1 (Chiang et al., 2013). Therefore, RvD1 presents an active role in inducing Nrf2 that will orchestrate antioxidant responses.

In conclusion, the present study demonstrated for the first time, to our knowledge, that treatment with RvD1 inhibits oxidative and inflammatory damage induced by exposure to UVB irradiation in hairless mice. RvD1 inhibited the inflammatory cell counts in the skin, and since the migration and activation of inflammatory cells were damped by reducing cytokine production and inducing antioxidant and anti-inflammatory genes, as a result, RvD1 protected the skin from UVB irradiation-induced tissue alterations such as collagen degradation. Therefore, the present results suggest RvD1 as a potential therapy to control UVB-induced skin inflammation- and oxidative stress-related alterations.

## Author contributions

PS, CM, RM, VF, TC, IP, and AB performed experiments. PS, RM, WV, and RC analyzed and interpreted data. WV and RC were responsible for conception and design of the study. PS organized the database. JV, SG, MB, WV, and RC provided research mentorship, supervision, received grants and provided essential reagentes. PS, RM, WV, and RC wrote the manuscript. All authors contributed to manuscript revision, read and approved the final version.

### Conflict of interest statement

The authors declare that the research was conducted in the absence of any commercial or financial relationships that could be construed as a potential conflict of interest.
